# Dermoscopy of oral mucocele: three types of extravasation mucoceles

**DOI:** 10.3906/sag-1907-56

**Published:** 2020-02-13

**Authors:** Erhan AYHAN, Serdar Ferit TOPRAK, Şeyhmus KAYA, Şerike AKKAYNAK

**Affiliations:** 1 Department of Dermatology, University of Health Sciences Gazi Yaşargil Training and Research Hospital, Diyarbakır Turkey; 2 Department of Otorhinolaryngology, University of Health Sciences Gazi Yaşargil Training and Research Hospital, Diyarbakır Turkey; 3 Department of Pathology, University of Health Sciences Gazi Yaşargil Training and Research Hospital, Diyarbakır Turkey; 4 Department of Pathology, Selahaddin Eyyubi State Hospital, Diyarbakır Turkey

**Keywords:** Mucocele, cyst, neoplasms, dermoscopy

## Abstract

**Background/aim:**

Dermoscopy is a diagnostic tool that assists in imaging the epidermis and dermis. Although it has also started to be used to diagnose nonmelanocytic lesions recently, it has not been tested much on oral mucosal masses, such as oral mucoceles. This study aimed to investigate whether dermoscopy is a valuable tool in diagnosing oral mucoceles.

**Materials and methods:**

In this study, the clinical and dermoscopic features of 21 oral mucocele lesions of 21 patients (11 females, 10 males) aged between 6 and 38 years who were confirmed histopathologically were evaluated.

**Results:**

Of the lesions studied, 95.2% (20) were extravasation and 4.8% (1) were retention mucoceles. The nonvascular structures were determined as white areas (61.9%, 13), erythema (57.1%, 12), purplish-gray background (52.3%, 11), ulcer (30%, 8), yellowish-orange areas (23.8%, 5), crust (14.2%, 3), starburst pattern (0.95%, 2), and bleeding (0.47%, 1). Dermoscopically, 40% of extravasation mucoceles were classified as type 1 (8 patients), 25% as type 2 (5 patients), and 35% as type 3 (7 patients).

**Conclusion:**

We concluded that there are 3 types of extravasation mucoceles dermoscopically and clinically, and these types may be stages of transition between each other.

## 1. Introduction

Oral mucoceles are the most frequently observed benign lesions of the minor salivary gland and are formed as a result of any mechanical trauma on the discharge duct of the salivary gland. There are 2 types of mucoceles: extravasation mucocele (EM) and retention mucocele (RM) [1,2]. Extravasation mucoceles emerge as a result of the extravasation of salivary gland secretions from the salivary gland duct into the soft tissues around the gland. Meanwhile, the obstruction of the salivary gland ducts, which leads to the reduction or absence of glandular secretion, causes RM [3,4]. These are accepted as separate from each other since each has a unique pathogenesis and microscopic properties [5]. While the lesions are more common in the internal part of the lower lip, they may also be present on the buccal mucosa, tongue, and floor of the mouth [6–8]. 

Dermoscopy is a diagnostic tool that is used to identify both melanocytic and nonmelanocytic lesions. Although it is generally used to diagnose melanocytic skin diseases, it has also started to be used to diagnose nonmelanocytic skin diseases in recent years [9]. There are very few dermoscopic publications concerning oral mucosal diseases [10,11]. This study aimed to investigate the dermoscopic and clinical characteristics of histopathologically verified mucoceles.

## 2. Materials and methods

An ethical committee decision was obtained for the study. Twenty-one patients (11 female, 10 male) of ages ranging from 6 to 38 years (mean 20.38), who applied at the dermatology department as outpatients and received a mucocele diagnosis histopathologically, were included in the study. The lesions were totally excised by the otorhinolaryngologist after dermoscopic evaluation. This study covers 3 stages: the dermatological and dermoscopic examination of the lesions (Dermatoscope Delta 20; Heine, Herrsching, Germany; Handyscope Fotofinder Systems), photographing the findings, and evaluating them. All lesions in the study were photographed macroscopically (at least 3) and by the hand dermatoscope (at least 10), and the data were recorded. The structures that were classified as vascular and nonvascular were identified dermoscopically. The contact plate was washed with physiological saline prior to taking the dermoscopic images in order to increase the image quality and the visibility of the structures. The pressure on the lesion was relieved in order to prevent the vascular structures from collapsing.

Scoring for connective tissue increase, inflammation, and vascular proliferation (0: absent, 1: mild, 2: moderate, 3: severe) was performed. Epithelial thickness and mucus spread were measured microscopically. The amount of mucin was evaluated by 2 pathologists by calculating the area at 40× magnification. The base and surface characteristics of the lesions were also evaluated.

All patient data were uploaded to SPSS 17.0 for Windows (SPSS Inc., Chicago, IL, USA). The Mann–Whitney U test and Pearson chi-square test were used to compare the parameters. A P-value of <0.05 was accepted as indicating statistical significance.

## 3. Results

**Figure 1:**
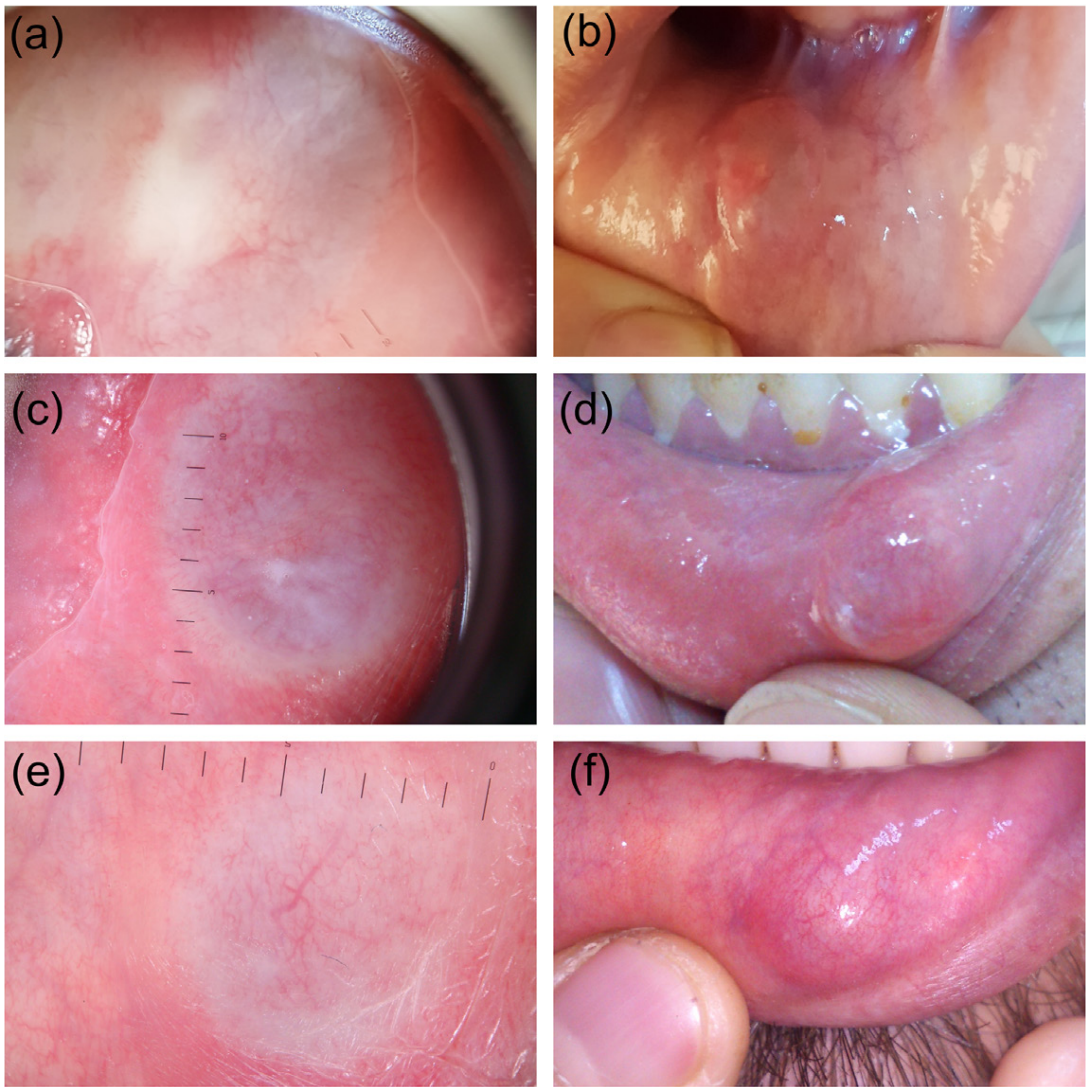
Extravasation mucocele type 1 lesions. Dermoscopy shows a purplish-gray background (a, c, e), reticularly branching vessels (a, c, e), yellowish-orange area (a), and white area (c). Macroscopic pictures (b, d, f) of these patients have nodular appearance.

**Figure 2:**
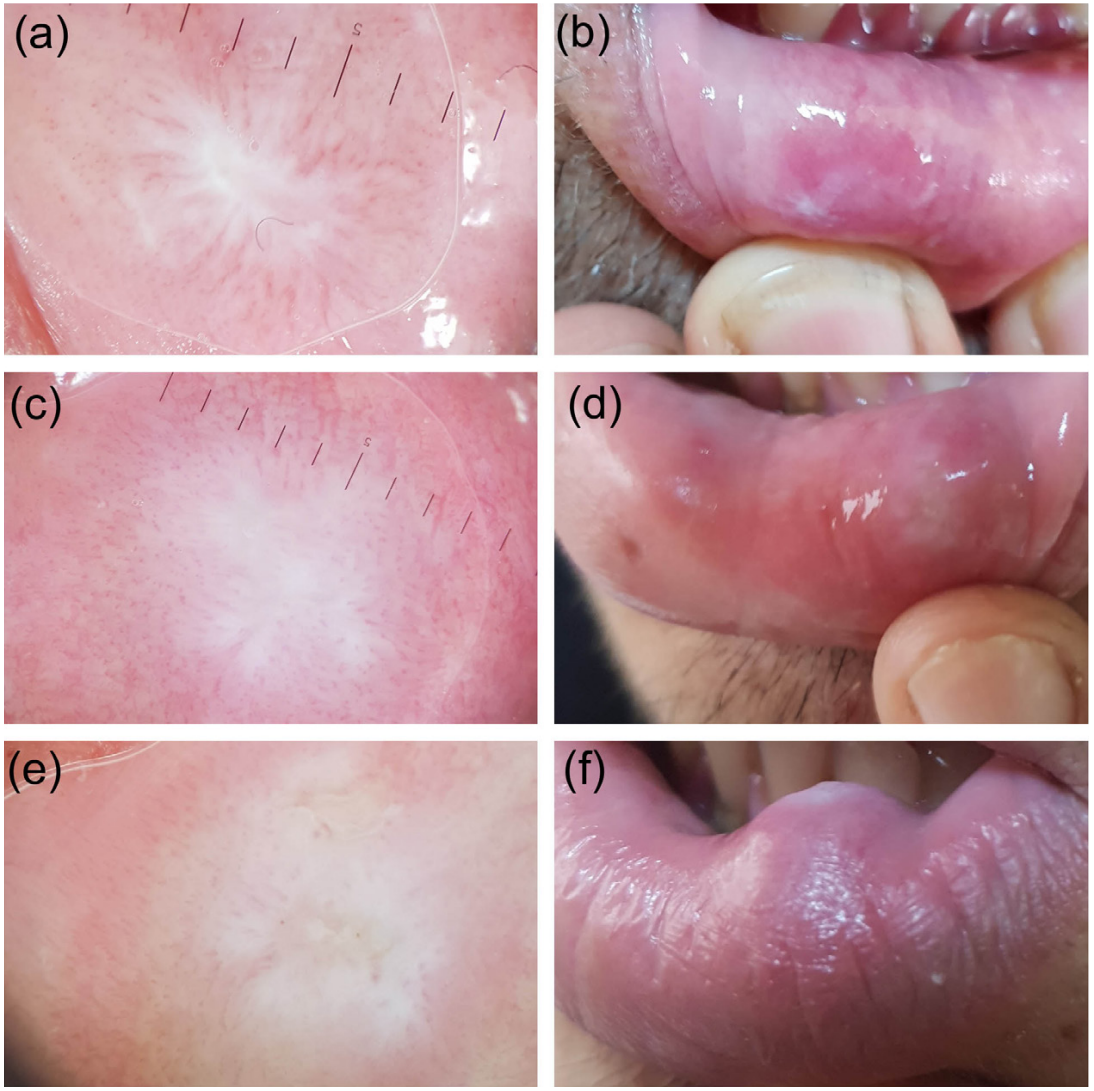
Extravasation mucocele type 2 lesions. In dermoscopy, the hairpin-shaped vessels (a, c, e) surrounding the white area (a) in the starburst style and white areas (c,e) on the erythematous background extend into the center. In macroscopic pictures, there are nodular lesions (b, d, f) with white surface.

**Figure 3:**
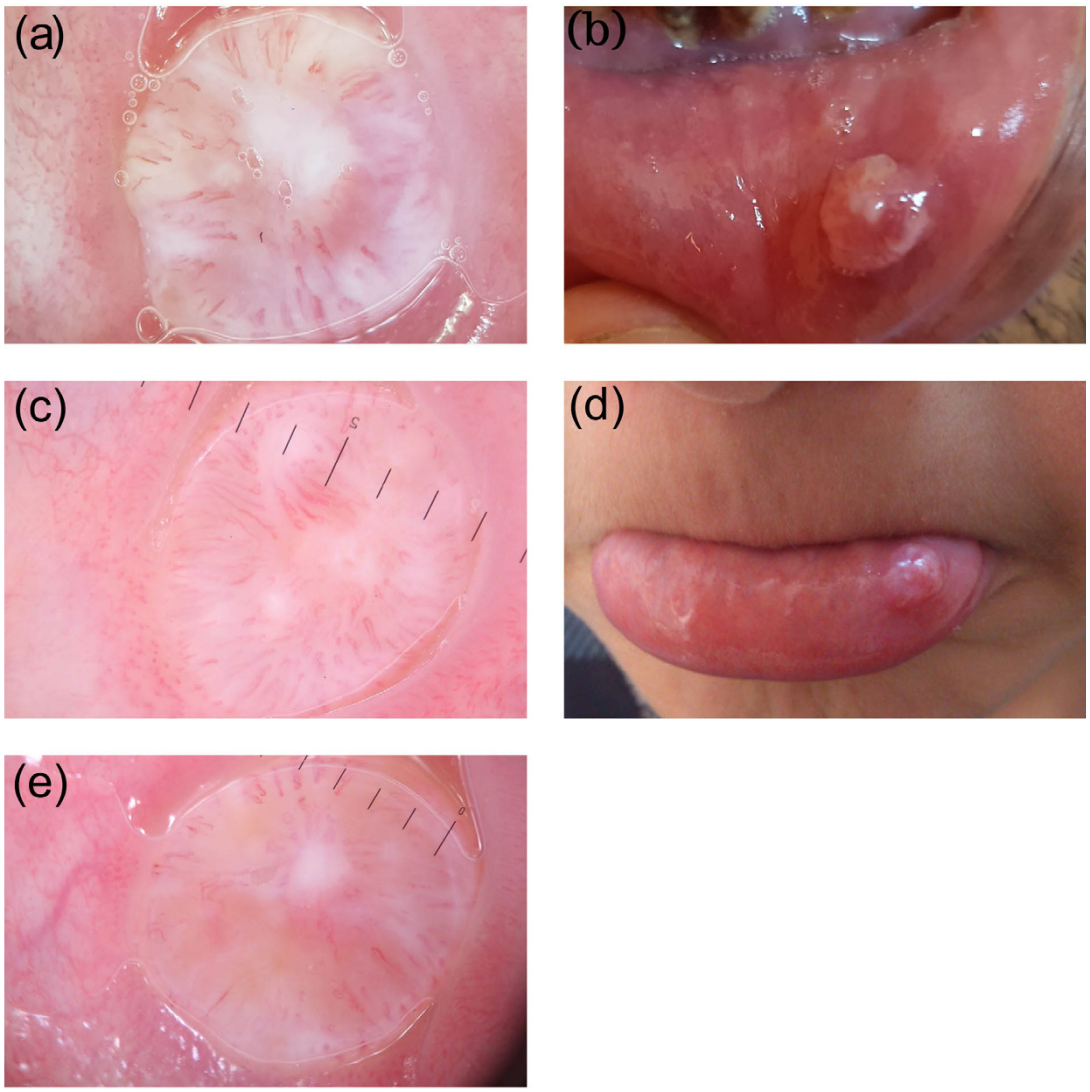
Extravasation mucocele type 3 lesions. Dermoscopically, white areas (a, c, e), yellowish-orange areas (a, c, e), and hairpin-shaped vessels (a, c, e) on the erythematous ground in all lesions show orientation towards the center. In macroscopic pictures (b, d) of image a and c, dome-shaped lesions are seen with a sharp and irregular border. There is not a macroscopic picture of image e.

The study included 21 lesions of 21 patients, the diagnoses of which were verified histopathologically. The patients in the group were 52.4% (11) female and 47.6% (10) male. The patients’ ages ranged from 6 to 38 years, with an average of 20.38 ± 09.07. Of the lesions, 95.2% (20) were extravasation and 4.8% (1) were retention mucoceles. All lesions were localized on the lower lip mucosa. The starting time of the lesions varied between 1 week and 12 months. The lesion diameters varied in the range of 4–15 mm, and the average diameter was 7.47 mm (±2.96). The nonvascular structures were determined as white areas (61.9%, 13), erythema (57.1%, 12), purplish-gray background (52.3%, 11), ulcer (30%, 8), yellowish-orange areas (23.8%, 5), crust (14.2%, 3), starburst pattern (0.95%, 2), and bleeding (0.47%, 1). The vascular structures were defined as hairpin-like vessels (57.1%, 12), branching vessels (42.8%, 9), dot vessels (33.3%, 7), and comma-like vessels (0.47%, 1). The lesions were described as erythematous (57.1%, 12) or purplish-gray background (52.3%, 11) based on their general colors. Two lesions were defined as both erythematous and purplish-gray background. While 47.6% (10) of the lesions were nodule and 52.3% (11) were dome-shaped, based on their base properties, 85.7% (18) had a smooth surface and 14.2% (3) had a lobular surface based on their surface properties. Extravasation mucoceles were divided into 3 types based on their clinical dermoscopic characteristics, such as color, base, and surface, and dermoscopic characteristics, such as vascular and nonvascular structures. The lesions defined as type 1 (Figure 1) were those with a purplish-gray background, soft, nodular, with smooth surfaces, and regular and uncertain borders, and were dermoscopically accompanied by reticular branching vessels. The lesions defined as type 2 (Figure 2) were those which were erythematous, soft, nodular, with smooth surfaces, and regular and uncertain borders, and were dermoscopically accompanied by hairpin-like vessels. The lesions defined as type 3 (Figure 3) were those which were erythematous, firm, dome-shaped, with sharp and irregular borders, and with smooth surfaces (rarely lobular surfaces), and were dermoscopically accompanied by hairpin-like vessels. We observed that the clinical characteristics in type 2 resembled type 1, while the dermoscopic characteristics were more similar to type 3. In addition, while the mucin material was drained by puncture in types 1 and 2, there was no mucin material drainage in type 3.

The mean age of patients with type 1 (23.5) and type 2 (25) lesions was higher than that of patients with type 3 (12.57) lesions (P = 0.016). A significant relationship could not be identified between the lesion diameters and types. The purplish-gray background and reticular branching vessels observed in type 1 lesions were statistically significant compared to other types (P = 0.001 and P = 0.002, respectively:). In types 2 and 3, the detection of erythema and hairpin-like vessels were significant compared to type 1 (P = 0.002 and P = 0.008, respectively). Yellowish-orange areas observed in type 3 were statistically significant compared to other types (P = 0.036). No significant relationship could be determined between the white areas and the 3 clinical types. However, they were identified more in types 2 and 3.

**Figure 4:**
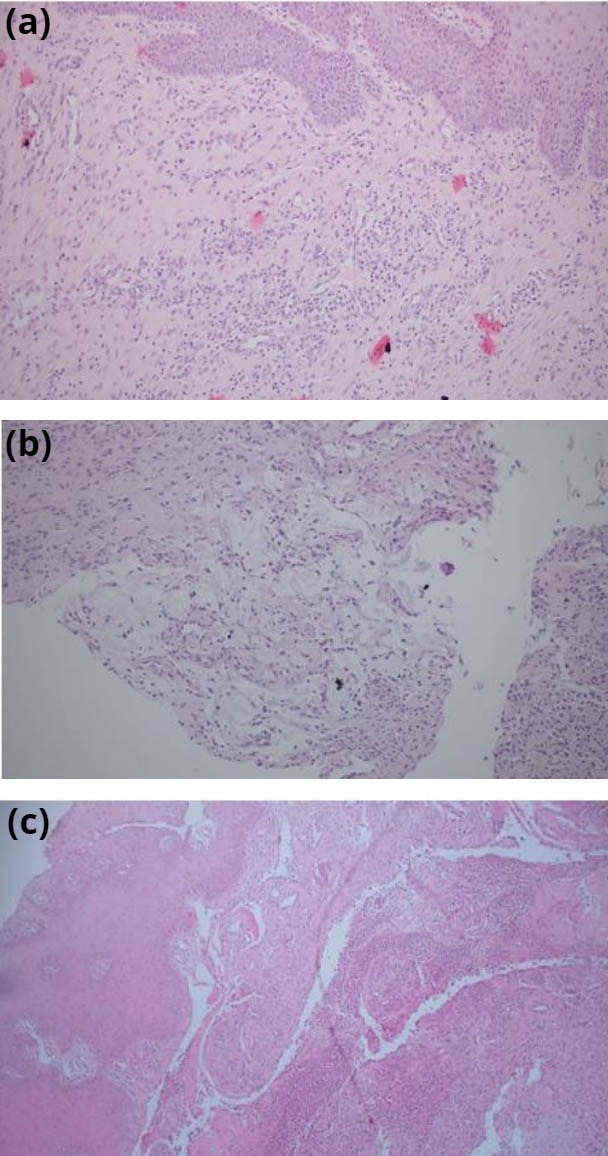
Type 1. Irregular acanthotic stratified squamous epithelium, scattered minimal mucoid accumulation in the epithelial tissue, moderately mixed type inflammation, and proliferation in vascular structures (H and E, ×100). (b). Type 2. Focal area of mucoid material in a scattered manner, mild to moderate inflammation, and proliferation in vascular structures (H and E, ×100). (c). Type 3. Stratified squamous epithelium, separation in subepithelial tissue, mild type inflammation, vascular proliferation, and mucoid material in heterogeneous distribution (H and E, ×100).

We observed more mucin at a statistically significant level in EM types 1 and 2 than type 3 when compared histopathologically, even after there has been a certain amount of material lost by puncture (P = 0.023, Table 1). A significant relationship between epithelium thickness, vascular proliferation, increased connective tissue, and inflammation could not be determined between the types (Figure 4). However, the epithelium thickness was less in retention mucoceles compared to EM.

**Table 1:** General clinic and histopathological features of extravasation mucocele.

Clinic and histopathological features of extravasation mucocele	Type 1 (n = 8)	Type 2 (n = 5)	Type 3 (n = 7)	P value
Sex (female/male)	4/4	2/3	5/2	0.053
Mean age of patients (years)	23.5	25	12.57	0.016
Number of patients (n/%)	8/40	5/25	7/35	0.239
Duration of lesions (month)	8.8	5.2	6.5	0.225
Puncture and mucin drainage	Yes (8/8)	Yes (5/5)	No (0/7)	0.000
Smooth surface	7/8	5/5)	5/7	0.380
Base	Nodular (3/8)	Nodular (2/5)	Dome-shaped (5/7)	0.371
Border of lesions	Regular andunclear (8/8)	Regular andunclear (5/5)	Irregular andsharply (7/7)	0.000
Consistency of lesions	Soft (8/8)	Soft (5/5)	Firm (7/7)	0.000
Histopathological feature Amount of mucin (mean/mm2)	0.312 mm2	0.287 mm2	0.135 mm2	0.023

While 5 of the patients described a trauma history leading to their lesions, 6 patients said that they had no trauma, and 10 said that they did not know how the lesions started. The clinical, histopathological, and dermoscopic features of EM types are shown in Tables 1 and 2.

**Table 2:** Dermoscopic features of extravasation mucocele.

Dermoscopic structures	Type 1 (n = 8)	Type 2 (n = 5)	Type 3 (n = 7)	P value
Purplish-gray background	8/8	1/5	1/7	0.001
Erythematous background	1/8	5/5	6/7	0.002
Branched vessels (reticular)	7/8	0/5	1/7	0.002
Hairpin-like vessels	1/8	4/5	6/7	0.008
Yellowish-orange areas	1/8	0/5	4/7	0.036
White areas	4/8	4/5	5/7	0.493
Dot vessels	1/8	5/5	1/7	0.002
Comma-like vessels	1/8	0/5	0/7	0.454
Ulceration	1/8	2/5	5/7	0.067
Crust	0/8	2/5	0/7	0.036
Bleeding	1/8	0/5	0/7	0,.454

## 4. Discussion

An oral mucocele is a widespread salivary gland lesion arising from mucus accumulation. It is observed most frequently on the lower lip, since the lower lip is more prone to trauma due to its anatomic localization [12]. A mucocele is clinically observed as asymptomatic vesicles and pink or bluish-colored bulla, and the dimensions vary from 1 mm to several centimeters [13]. It has the highest incidence of occurrence between ages 10 and 20 [14]. Mucoceles may appear as either extravasation or retention types [12]. EM is widespread in children, while RM is very rare. 

A retention mucocele emerges from the obstruction of the salivary duct by a sialolith or a scar in the duct, and the mucin is then surrounded by ductal epithelium. While RM is associated with the traumatic injury of the ductus, EM emerges from the extravasation of the saliva to the adjacent connective tissue areas [14]. 

Extravasation mucoceles undergo 3 developmental phases. The mucus infiltrates into the connective tissues from the mucus discharge duct in the first phase. In the next stage, the resorption phase, granuloma formation occurs due to a foreign substance reaction. In the final stage, a pseudocapsule forms around the mucosa (without epithelial lining) [3,4]. 

Dermoscopy is a valuable diagnostic tool for both melanocytic and nonmelanocytic lesions. To the best of our knowledge, dermoscopic profiles for oral mucocele lesions have not been previously reported in the English literature. 

In our study, 95.2% of the subjects (20 patients) had EM. Only one subject had RM. The studies regarding the incidence levels of mucoceles between sexes are controversial [15]. According to 1 study, incidence levels between men and women were not observed to be significant different [16]. In our study, 52.4% (11) of the patients were female and 47.6% (10) were male. There was no significant difference between the females and males.

Oral mucocele of the minor salivary gland is generally observed in young people. The peak age of the occurrence of this mucocele has been reported as 10–20 years. According to Liu et al., 43.7% of the total patients studied were 10–20 years of age and 37.5% of patients were under 10 years of age. [4]. Children under 15 years of age were 62.5% of patients studied. In our study, the ages of the patients ranged from 6 to 38. The average age was 20.38 ± 9.07, with 47.6% (10) of the subjects 20–30 and 19% (4) 10–20.

Diagnosis of mucoceles is principally based on clinical findings. The appearance of mucoceles is pathognomonic, and the location of the lesion, trauma history, rapid formation, changes in dimensions, bluish color, and texture are important factors that need to be taken into consideration prior to the final diagnosis [17]. The extravasation type is more widespread. This type may be misdiagnosed as traumatic fibroma when it does not have a soft texture and bluish color [1,2,18]. The displacement of the epithelium by fibrinopurulent membrane with dense chronic inflammatory cells may cause the surface of the lesion to appear yellowish [18]. According to the literature, classical mucoceles are known as bluish and soft [1,2,18]. Without this color and consistency, it is reported that it may be misdiagnosed [18]. In our study, bluish/purplish color was not observed in 60% of EMs (types 2 and 3), and soft consistency was not observed in 25% (type 3). We classified mucoceles that were evaluated as soft and bluish as type 1, and those with different clinical and dermoscopic appearances as type 2 and type 3. Type 1 mucocele had purplish color, reticular branching vessels, and material drainage by puncture. In type 2, erythema, hyperkeratotic white areas, unclear hairpin vessels, and puncture material drainage were observed. Type 3 showed erythema, yellow areas, and marked hairpin vessels. There was no material drainage by puncture. 

In most cases, particularly with type 3 lesions, the lesions begin as purplish and then grow and become habitually bitten, which causes the color to shift to white by creating hyperkeratosis like that found in type 2 lesions, and eventually turns into irregular and sharp confined lesions like those found in type 3.

With these different clinical and dermoscopic features, we think that EMs begin as type 1, known as the classical type, and then evolve into type 2, with white areas in the form of hyperkeratosis with recurrent trauma, and then evolve into type 3, with yellow color, a sign of the chronic appearance mentioned in the literature. 

The lesions in type 1 were soft, nodular, purplish-gray in color, and had uncertain and regular borders. The lesions in type 2 were soft, nodular, erythematous, and had uncertain and regular borders. The lesions in type 3 were firm, dome-shaped, erythematous, and had sharp and irregular borders. Furthermore, mucin material could be drained by puncture in types 1 and type 2.

The appearance of both EM and RM are clinically similar. Mucoceles appear as bluish, soft, and transparent cystic tumescences that frequently dissolve spontaneously. The blue color is associated with vascular congestion, cyanosis of the tissue at the top, and accumulation of fluid beneath. However, coloring may change depending on the size of the lesion, its closeness to the surface, and the flexibility of the tissue at the top [12]. The purplish-gray color observed in type 1 was associated with the excessive amount of mucoid material based on the pathological evaluations. Although epithelium thickness was increased in all types of EM, this thickness was less in RM compared to EM.

The term oral exophytic lesion is defined as pathological growth that bulges above the normal contours of the oral mucosa [19]. There are many basic mechanisms responsible for oral exophytic lesions such as hypertrophy, hyperplasia, neoplasia, and pooling of fluid [20]. This complicates approaching these lesions clinically [21,22]. According to the national epidemiological study of Zain et al., 26% of all lesions are exophytic lesions [21]. Exophytic lesions can be classified according to their surface texture (rough or smooth), base type (nodular, dome-shaped, sessile, or pedunculated), and consistency (firm, soft, cheesy, rubbery, or bony hard) [20,22,23]. While 47.6% (10) of the lesions are nodule-shaped and 52.3% (11) are dome-shaped based on their base characteristics, 85.7% (18) have smooth and 14.2% (3) have lobular surfaces based on their surface characteristics. In this study, 75% (6) of the lesions were nodular in EM type 1, 60% (3) were nodular in type 2, and 85.7% (6) were dome-shaped in type 3. When they were examined based on surface characteristics, all of types 1 and 2 were smooth, while 57.1% (4) were smooth and 42.9% (3) were lobular in type 3. 

In conclusion, this study is the first such dermoscopic study carried out on oral mucosa. Three clinical forms of EM were identified from clinical and dermoscopic perspectives. Lesions with purplish-gray background and reticular branching vessels were identified as type 1, with erythematous base and hairpin vessels as type 2, and with hairpin vessels on erythematous base and yellowish-orange areas as type 3. We believe that these types are stages of transition from type 1 to type 3.
